# Retrograde Stent Graft Placement for Chronic Total Occlusion of the Venous Anastomosis in a Forearm Loop Arteriovenous Graft: A Case Report

**DOI:** 10.7759/cureus.110422

**Published:** 2026-06-07

**Authors:** Sojiro Amamoto, Junichi Yoshida, Yoshito Shogakiuchi, Yoshiaki Kinoshita, Katsuhiko Takenaka

**Affiliations:** 1 Department of Vascular Surgery, Shigematsu Clinic, Fukuoka, JPN; 2 Department of Vascular Surgery, Shinai Clinic, Fukuoka, JPN; 3 Department of Cardiology, Shinai Clinic, Fukuoka, JPN; 4 Department of Nephrology, Shigematsu Clinic, Fukuoka, JPN; 5 Department of Cardiology, Shigematsu Clinic, Fukuoka, JPN

**Keywords:** arteriovenous graft, chronic total occlusion (cto), hemodialysis vascular access, retrograde approach, stent graft

## Abstract

Chronic total occlusion (CTO) at the venous anastomosis of an arteriovenous graft (AVG) remains difficult to treat endovascularly, particularly when an antegrade approach via the graft is unsuccessful. We report the case of a 90-year-old woman receiving maintenance hemodialysis through a left forearm loop AVG who developed progressive access dysfunction due to a CTO extending from the venous anastomosis to the downstream basilic vein. Because antegrade recanalization had failed and surgical revision was considered less desirable given her advanced age, frailty, and fragile skin condition, an alternative endovascular strategy was selected. Preprocedural ultrasound confirmed a downstream basilic vein segment suitable for retrograde puncture. Under ultrasound guidance, the downstream outflow vein was punctured, and a 6-Fr sheath was inserted retrogradely. After successful retrograde wire crossing of the occluded segment, cautious predilation was performed with a 4 × 40 mm balloon, followed by retrograde deployment of a 6 × 100 mm stent graft with overlap into the graft. Technical success was achieved without complications. The procedure was completed on an outpatient basis in 58 minutes. Postprocedural duplex ultrasound showed improved access hemodynamics, and the AVG remained functional without restenosis or reintervention at approximately six months of follow-up. This case suggests that retrograde stent graft placement via the downstream outflow vein may be a feasible salvage option in carefully selected patients with venous anastomotic CTO of an AVG when an antegrade approach is unsuccessful.

## Introduction

Venous anastomotic lesions are the most common cause of dysfunction in arteriovenous grafts (AVGs) used for hemodialysis and are associated with high rates of restenosis after percutaneous transluminal angioplasty alone [1,2]. In particular, chronic occlusion at the venous anastomosis remains challenging for endovascular treatment because of fibrotic changes, elastic recoil, and difficulty in guidewire crossing [3,4]. Although an antegrade endovascular approach via the graft is generally considered the first-line strategy, recanalization may fail in cases of a chronic total occlusion (CTO). In such situations, alternative approaches are required to salvage the access while avoiding surgical revision, especially in elderly or high-risk patients.

Stent grafts have been shown to improve patency in venous anastomotic lesions of AVGs and are now widely used in cases of recurrent stenosis or thrombosis [1,5-7]. However, to the best of our knowledge, there have been no prior reports clearly describing retrograde stent graft placement via the downstream native outflow vein for chronic occlusion at the venous anastomosis of an AVG. In carefully selected patients, especially frail elderly patients in whom conventional antegrade treatment has failed, and surgical revision is undesirable, such an approach may offer a practical option for preserving dialysis access.

We report a case of chronic occlusion at the venous anastomosis of a forearm AVG that was successfully treated with retrograde stent graft placement via the downstream native outflow vein after an unsuccessful antegrade approach.

## Case presentation

**Figure 1:**
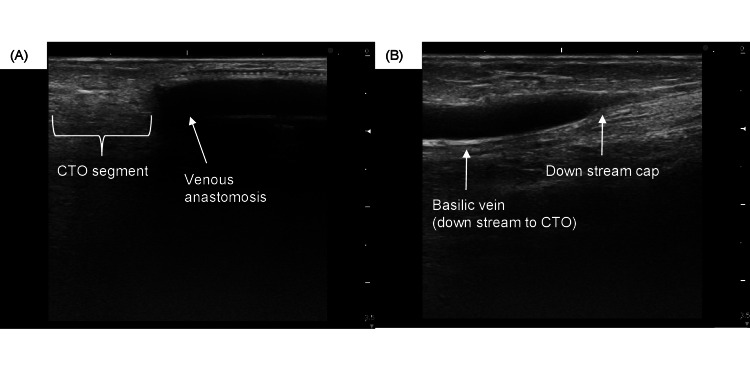
Preprocedural ultrasound (B-mode). (A) Long-axis view of the venous anastomosis of the left forearm loop arteriovenous graft (AVG). The upstream cap of the chronic total occlusion (CTO) segment could not be clearly identified. (B) Long-axis view of the downstream outflow vein (basilic vein) beyond the CTO. The downstream cap was visible, and the intraluminal echoes were relatively hypoechoic. The vein diameter was approximately 5 mm, and the depth from the skin was 6-7 mm, indicating that ultrasound-guided retrograde puncture and sheath insertion were technically feasible.

A 90-year-old woman had been undergoing maintenance hemodialysis for three years using a left forearm loop AVG, which had been in use for two years. Balloon angioplasty had been performed for stenosis extending from the venous anastomosis to the downstream basilic vein approximately two years before the present procedure. During follow-up, ultrasound examination revealed a CTO measuring approximately 7 cm in length, extending from the venous anastomosis to the downstream basilic vein (Figure 1A and B).

**Figure 2:**
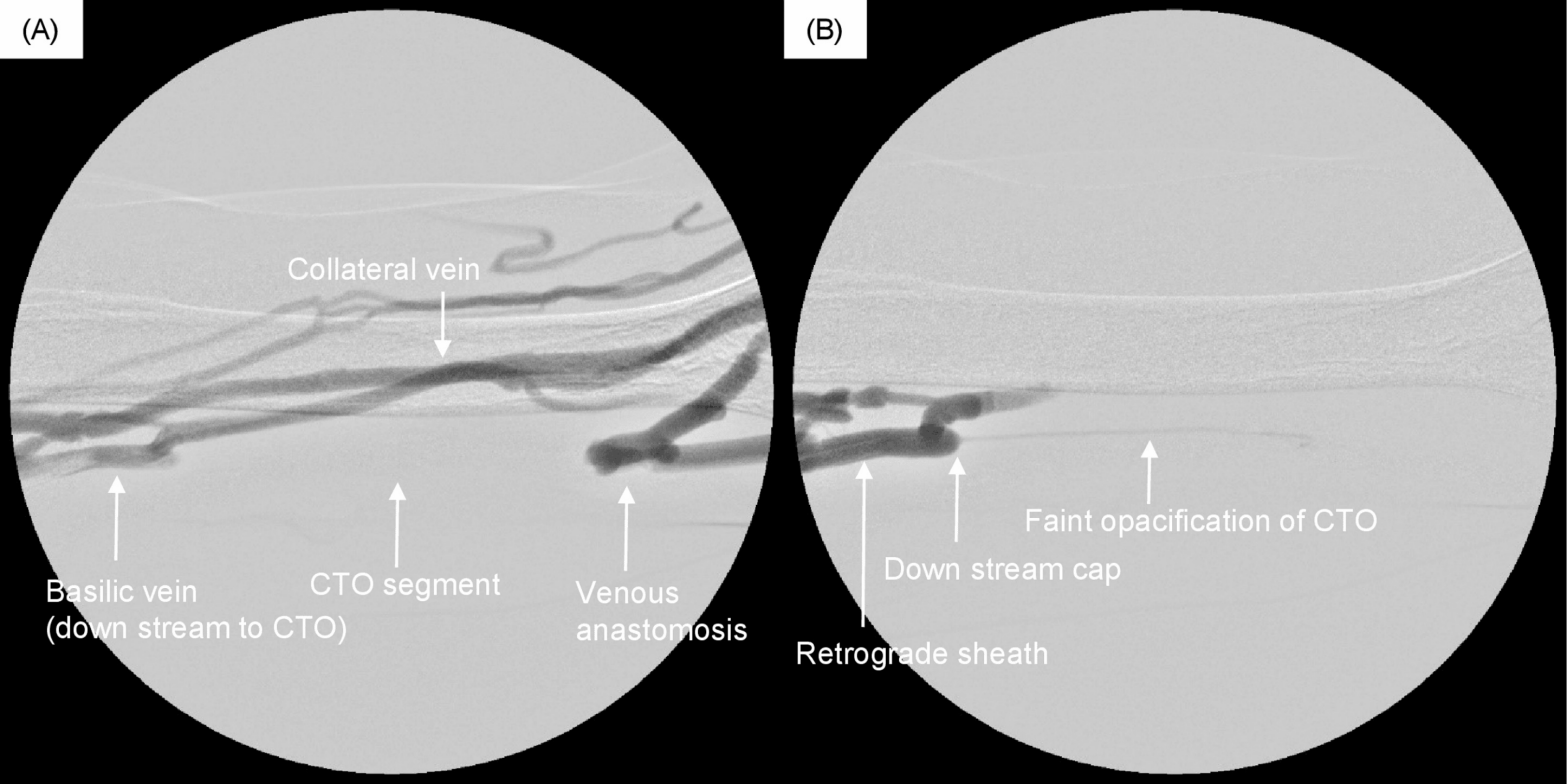
Intraprocedural angiography. (A) Antegrade angiography demonstrated a CTO at the venous anastomosis of the left forearm loop AVG. The downstream native outflow vein remained patent via collateral venous pathways. The CTO length was approximately 7 cm. (B) Retrograde angiography via the retrograde sheath showed faint opacification of the CTO segment through the downstream cap, supporting the feasibility of retrograde lesion crossing.

Although access blood flow was maintained through collateral circulation via the median cubital vein draining into the brachial vein, progressive access dysfunction raised concern for further deterioration of dialysis access function and prompted consideration of additional intervention (Figure 2).

Surgical revision was considered; however, given the patient’s advanced age, frailty, and fragile skin condition, a less invasive strategy was deemed preferable. A standard antegrade endovascular approach via the graft had previously been attempted using a 6-Fr sheath. However, the guidewire could not cross the chronic occlusion. Sharp recanalization using a metal introducer under ultrasound guidance was also attempted but was unsuccessful and resulted in a limited perivascular hematoma [8]. As access blood flow was preserved through collateral circulation, the procedure was discontinued to avoid further complications.

Preprocedural ultrasound examination confirmed the presence of an adequate downstream native basilic vein segment suitable for retrograde sheath insertion. In addition, we confirmed that the target vein was not excessively deep and did not course posterior to the brachial artery, which would make puncture technically difficult or unsafe (Figure 1B) [9].

Surgical AVG revision was also planned as the final treatment option if the retrograde stent graft approach proved unsuccessful.

Because the upstream cap of the chronic occlusion (graft side) was difficult to penetrate using an antegrade approach, a retrograde strategy via the downstream outflow vein was selected. By analogy to CTO interventions, in which crossing from the downstream cap may be more feasible, we anticipated that retrograde crossing from the downstream outflow vein could facilitate lesion traversal. In our case, the downstream occlusion segment appeared less echogenic on ultrasound compared with the upstream cap, which supported this strategy [10].

**Figure 3:**
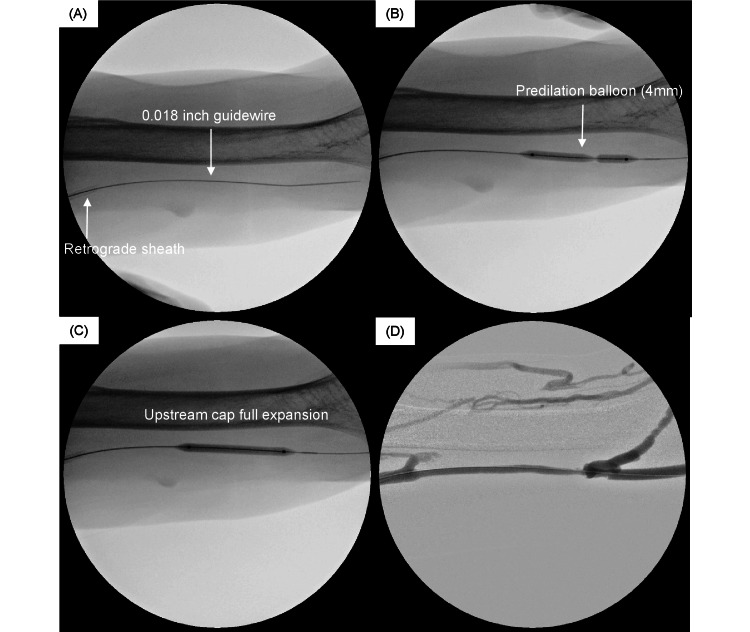
Wire crossing and predilation of the CTO (procedural steps). (A) Fluoroscopic image showing retrograde passage of a 0.018-inch guidewire across the CTO, advanced up to the arterial anastomosis of the arteriovenous graft (AVG). (B) Predilation of the CTO via the retrograde sheath using a 4-mm balloon catheter. The balloon engaged the upstream cap. (C) Predilation continued, and the balloon ultimately achieved full expansion. (D) Angiography after predilation via the retrograde sheath demonstrating restoration of flow through the previously occluded segment, indicating successful recanalization before stent graft placement. CTO: chronic total occlusion.

Under ultrasound guidance, the native basilic vein downstream from the occlusion was punctured, and a 6-Fr sheath was inserted retrogradely [9]. A 0.018-inch V-18 Control guidewire (Boston Scientific Corporation, Marlborough, Massachusetts) was advanced retrogradely and successfully crossed the occluded segment (Figure 3A). Because the occluded venous segment showed severe negative remodeling and marked luminal narrowing, consistent with chronic occlusion, predilation with a balloon matching the stent graft diameter was considered to carry a risk of vessel injury. Therefore, predilation was intentionally performed using a smaller 4 × 40 mm balloon catheter (BRAVUS; Boston Scientific Corporation, Marlborough, Massachusetts) to minimize the risk of vascular injury while creating sufficient space for stent graft delivery (Figure 3B-D).

**Figure 4:**
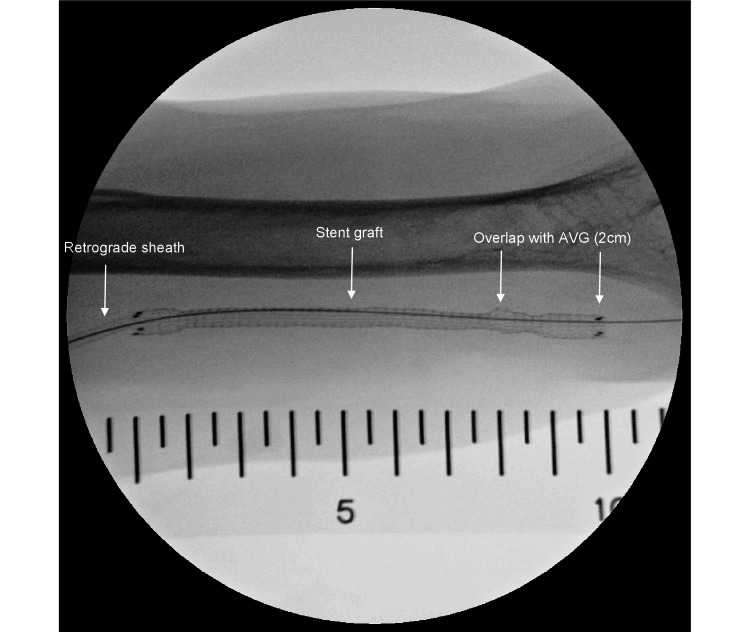
Retrograde deployment of a stent graft (before postdilation). A stent graft was advanced through the retrograde sheath and deployed to fully cover the recanalized CTO segment. The stent graft overlapped the AVG by 2 cm, providing a secure landing zone and fixation within the graft. CTO: chronic total occlusion.

Subsequently, a 6 × 100 mm Viabahn stent graft (W. L. Gore & Associates, Flagstaff, Arizona) was deployed retrogradely, covering the entire occluded segment (Figure 4).

The upstream end of the stent graft overlapped approximately 2 cm into the graft to ensure secure fixation. Because retrograde deployment results in an atypical deployment orientation compared with standard antegrade technique, we intentionally selected a more generous graft overlap to enhance anchoring and procedural safety [6].

After stent graft deployment, postdilation was performed using a 6 × 40 mm balloon catheter (Athletis; Boston Scientific Corporation, Marlborough, Massachusetts) to achieve adequate apposition.

**Figure 5:**
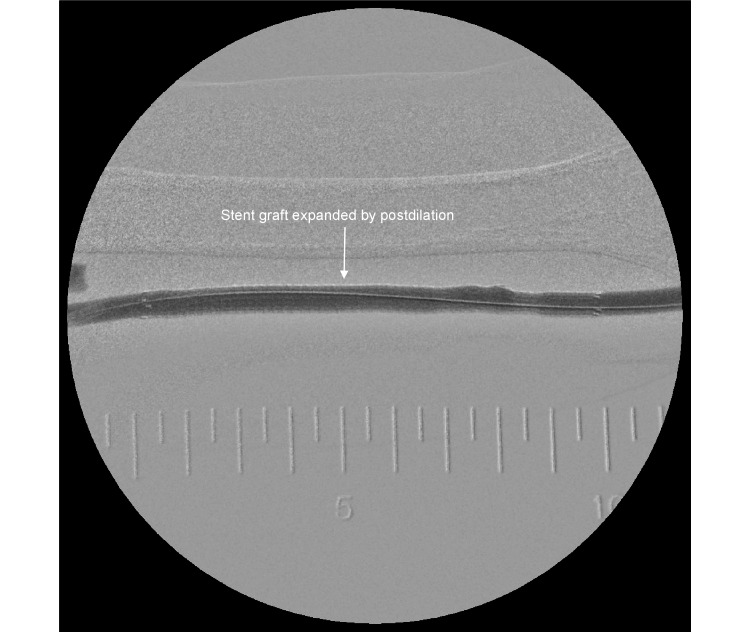
Final angiography after stent graft postdilation. The stent graft was fully expanded and maintained the lumen across the treated CTO segment. Collateral venous flow resolved on the final angiogram, suggesting improved outflow through the recanalized main channel. CTO: chronic total occlusion.

After successful retrograde predilation and recanalization of the chronic occlusion, conversion to an antegrade approach for conventional stent graft deployment was considered an alternative strategy. However, stent graft migration toward the central vein is a potentially serious complication during stent graft placement for AVG lesions. In this case, continuing the procedure via the retrograde sheath was considered advantageous because the retrograde sheath and guidewire could act as a mechanical barrier, potentially preventing migration toward the central vein of the stent graft in the event of inadvertent displacement. Based on this intra-procedural risk assessment, stent graft deployment was intentionally performed via the retrograde approach [11,12]. Technical success was achieved without complications (Figure 5).

The total procedure time was 58 minutes, and the procedure was completed on an outpatient basis. Duplex ultrasound showed improvement in access hemodynamics, with the resistance index decreasing from 0.67 preprocedurally to 0.62 postprocedurally and the access flow volume increasing from 479.9 to 660.1 mL/min [9]. At approximately 10 months of follow-up, the AVG has remained functional without restenosis and without the need for reintervention.

## Discussion

Chronic occlusion at the venous anastomosis of an AVG remains a challenging condition for endovascular treatment [1,3,4]. This case suggests that retrograde stent graft placement via the downstream native outflow vein may be a feasible salvage option in carefully selected patients when an antegrade approach fails and the downstream vein is accessible [9,10].

Careful ultrasound-guided access, including assessment of the vein depth and its relationship to the brachial artery, and adequate landing zones are essential to ensure procedural safety and device stability [9]. This approach may be less suitable in obese patients or when the basilic vein is deeply located, because puncture, sheath manipulation, and hemostasis can become more difficult. In the present case, preprocedural ultrasound confirmed that the target vein was not excessively deep and did not course posterior to the brachial artery, supporting the feasibility of retrograde sheath placement [9].

Compared with surgical revision, this endovascular approach may preserve native venous resources by avoiding extensive venous dissection or sacrifice, which is particularly advantageous in patients with limited future access options. In the present case, this also had practical clinical value because dialysis access could be preserved without surgical revision in a frail elderly patient.

Another possible strategy after retrograde wire crossing and predilation would be to externalize the wire using a pull-through technique and then deploy the stent graft antegradely from the graft side. We considered this option; however, in this case, continued use of the retrograde sheath was favored because it could help maintain control during deployment and potentially reduce the risk of migration toward the central vein [11,12].

In addition, if the stent graft is excessively long or migrates proximally during retrograde deployment, there is a potential risk of partial opening within the sheath or malposition at the venous exit site. Careful device-length selection, secure graft overlap, and meticulous sheath control are therefore important [6,11,12].

The Viabahn endoprosthesis features a contoured proximal edge that is intended to modify edge geometry and may influence local flow dynamics; it has also been discussed as a measure to mitigate edge infolding in some settings. We speculate that retrograde deployment may position this contoured edge at the downstream outflow-vein side in our configuration. This could be advantageous given that stent graft edge stenosis is a recognized mode of failure and is likely influenced by local hemodynamics. This hypothesis warrants further investigation [13,14].

At approximately 10 months of follow-up, the access has remained functional without reintervention. This short- to mid-term course appears broadly consistent with reported patency outcomes after stent graft placement for AVG-related lesions, although no formal comparison can be made from a single case. Therefore, this report should be interpreted as demonstrating technical and clinical feasibility in one carefully selected patient rather than long-term comparative efficacy.

## Conclusions

In this single case, retrograde stent graft placement via the downstream outflow vein helped salvage access while avoiding surgical revision. This approach may be considered in carefully selected patients, particularly those who are elderly or at high surgical risk and have a downstream outflow vein suitable for safe retrograde access.
